# Magnet‐Assisted Transanal Removal of a Rectal Foreign Body in a Child: A Case Report

**DOI:** 10.1002/ccr3.72236

**Published:** 2026-03-11

**Authors:** Daisuke Miyamoto, Shunsuke Nakamura, Tomoki Nakamori

**Affiliations:** ^1^ Japan Organization of Occupational Health and Safety Yokohama Rosai Hospital, Emergency Medicine Kanagawa Japan

**Keywords:** child, foreign bodies, magnets, minimally invasive surgical procedures, rectum

## Abstract

Management of rectal foreign bodies, uncommon in children, aims to achieve safe, minimally invasive removal while avoiding anesthesia or surgery whenever possible. We report a rare pediatric case of a rectal foreign body successfully removed using a magnet‐assisted transanal technique. To our knowledge, this technique has not been previously reported in pediatric patients. A 10‐year‐old girl with a developmental disability inserted an AAA battery into her rectum. She was asymptomatic on arrival. Pelvic radiography revealed a single cylindrical rectal foreign body. Considering the object type, short duration, and absence of perforation signs, transanal removal was planned. Manual grasping, endoscopic removal, and neuraxial or general anesthesia were considered difficult or overly invasive, so a magnet‐assisted technique was devised. A magnet placed between the double‐layered gloves of the examiner's index finger was gently advanced transanally, allowing atraumatic extraction. Magnet‐assisted transanal extraction is a simple and minimally invasive option for removing metallic rectal foreign bodies that respond to magnetic attraction. Further investigations are warranted to establish its safety and indications.

## Key Clinical Message

Magnet‐assisted transanal removal is a safe, minimally invasive option for magnetically reactive metallic rectal foreign bodies located within digital reach. It is essential to establish clear procedural limits and promptly escalate to alternative strategies to ensure patient safety.

## Graphical abstract

A 10‐year‐old girl with a developmental disability presented with a rectal foreign body after inserting an AAA battery. Pelvic radiography revealed a cylindrical object consistent with the battery. The foreign body was successfully removed transanally using a magnet‐assisted technique.

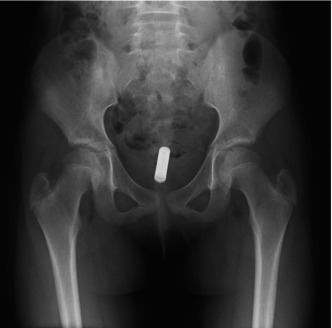

## 1 Introduction

Rectal foreign bodies can be encountered across all age groups but are relatively uncommon in pediatric patients [1, 2, 3]. In adults, rectal foreign bodies are often associated with voluntary transanal insertion, whereas in pediatric cases they may be related to curiosity, developmental disorders, or, less commonly, abuse [3, 4, 5]. Management strategies range from bedside transanal removal to endoscopic or surgical intervention, depending on object characteristics and any associated complications. Here, we report a rare pediatric case of a rectal foreign body involving an AAA battery, which was successfully removed using a magnet‐assisted transanal technique, thereby minimizing invasiveness and avoiding anesthesia. In pediatric patients, delayed diagnosis or inappropriate extraction attempts may increase the risk of rectal injury, highlighting the need for safe, simple, and child‐tailored removal strategies.

## 2 Case History/Examination

A 10‐year‐old girl with a history of type 1 diabetes mellitus and developmental disability presented to the emergency department approximately 2 h after inserting a single AAA battery into her rectum at home. She became anxious when the battery did not spontaneously pass and informed her mother, who then called emergency services. The patient did not have a history or suspicion of sexual abuse.

**FIGURE 1:**
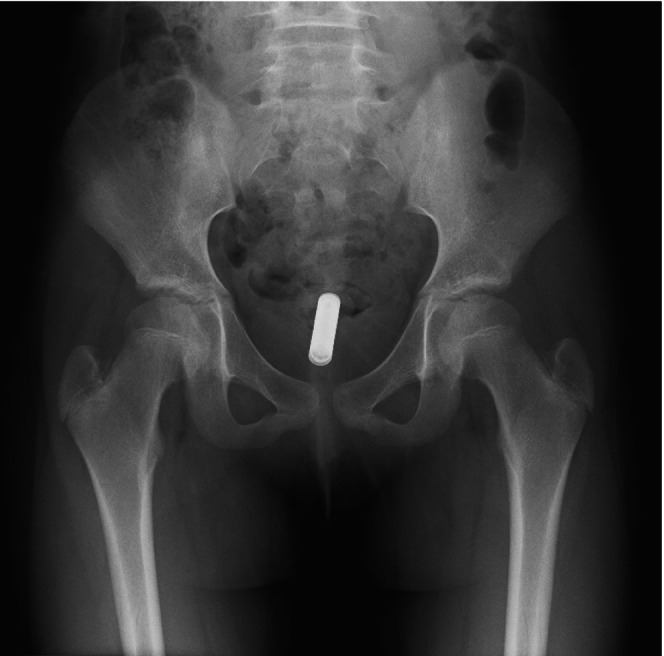
Pelvic X‐ray. The pelvic radiograph demonstrates a foreign body in the rectal region, measuring approximately 45 mm in length and 10 mm in width, consistent with the appearance of an AAA battery.

She was transported to our hospital, a tertiary emergency and critical care center, rather than a dedicated pediatric hospital. On arrival, the patient was ambulatory and hemodynamically stable, with the following vital signs: oxygen saturation, 98% on room air; respiratory rate, 20 breaths/min; pulse, 63 beats/min; blood pressure, 114/76 mmHg; and temperature, 36.7°C. She denied abdominal and anal pain. Abdominal examination was unremarkable. Digital rectal examination was initially deferred to avoid pushing the object proximally. Pelvic radiography revealed a single cylindrical foreign body, approximately 45 mm in length and 10 mm in width, in the rectum, consistent with an AAA battery (Figure 1).

## 3 Differential Diagnosis, Investigations and Treatment

Given the absence of symptoms, short interval since insertion, and object type, the risk of perforation was considered low, and transanal removal without computed tomography (CT) was planned. Due to the patient's age, limited cooperation, and small anal diameter, manual grasping without anesthesia was predicted to be difficult, and removal using endoscopy or anesthesia was considered overly invasive. Inspired by magnetic devices used for the removal of gastrointestinal foreign bodies, magnet‐assisted extraction was performed.

**FIGURE 2:**
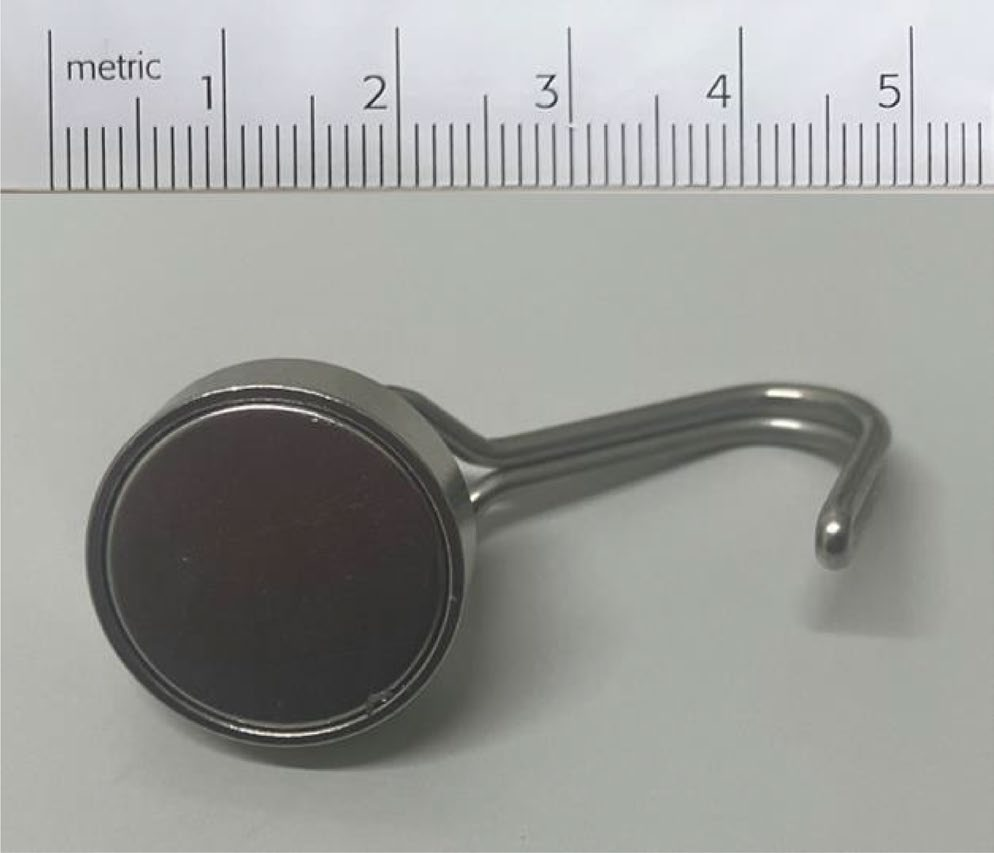
A commercially available neodymium magnet, approximately 20 mm in diameter with a hook, used in the extraction procedure.

**FIGURE 3:**
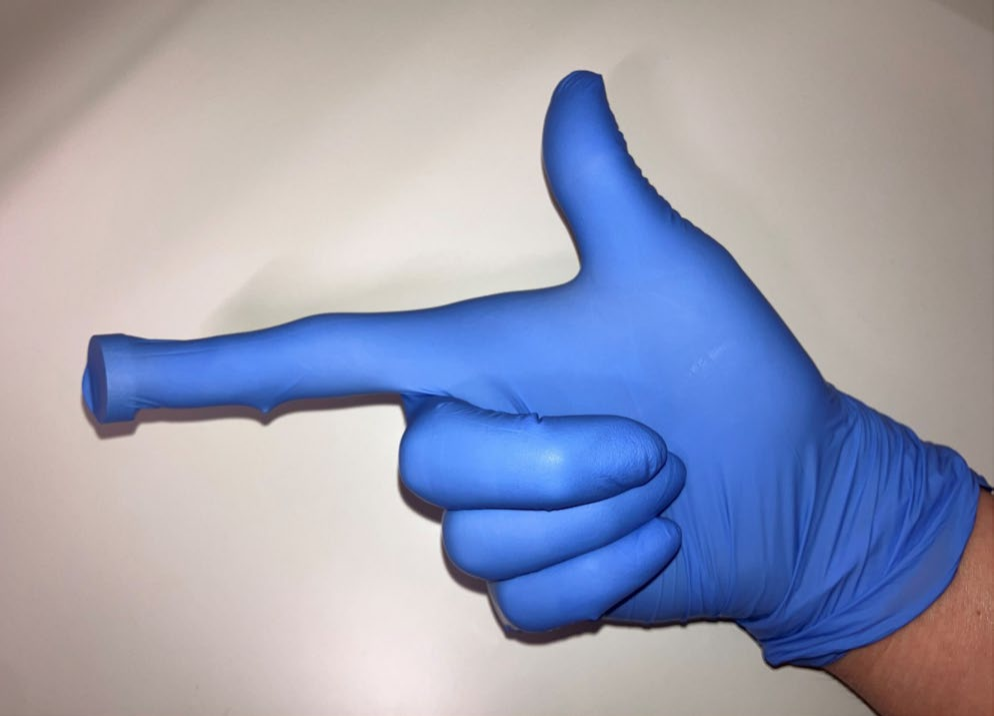
Schematic illustration of the extraction method. Double gloves were worn, and a magnet was placed between the outer and inner gloves at the tip of the index finger.

A commercially available neodymium magnet, approximately 20 mm in diameter, demonstrated sufficient attraction to an identical AAA battery during preprocedural trial (Figure 2). The procedure was performed by a physician board‐certified in both emergency medicine and pediatrics. The examiner wore double gloves and placed the magnet between the two glove layers on the index finger (Figure 3). After explaining the procedure and obtaining parental consent, the patient was positioned supine in a semi‐lithotomy position, and lidocaine gel was used for lubrication. The index finger was gently inserted transanally, the battery was palpated, and the magnetic contact was felt. Next, the battery was slowly and atraumatically withdrawn.

No signs of bleeding were present on the finger or the battery. Post‐extraction rectal examination revealed no obvious mucosal injury, and external genital examination was unremarkable. The patient was discharged with instructions to return in case of bleeding, abdominal pain, or other symptoms, and the family was advised on preventing recurrence.

## 4 Conclusion and Results (Outcome and Follow‐Up)

The battery was successfully extracted without anesthesia or complications. No rectal mucosal injury or delayed adverse events were observed. The patient was contacted by telephone 7 days after discharge, and no symptoms, including bleeding, abdominal pain, or anal discomfort, were reported. Since the patient was already under medical follow‐up for a developmental disability, an additional psychiatric consultation was deemed unnecessary at that time.

## 5 Discussion

Management principles for rectal foreign bodies in pediatric cases, which are rare, are primarily extrapolated from adult cases. In an analysis of a national emergency department database in the United States, Fischer et al. identified approximately 150 cases of rectal foreign bodies in children aged ≤ 12 years over a 10‐year period [4]. This indicates that such cases are encountered in emergency settings, although they are uncommon. The primary goals include exclusion of perforation, accurate localization, and selection of the least invasive and most effective method of removal. Therefore, CT should be considered in cases with unclear object characteristics, abdominal pain, systemic instability, or suspected complications. In this case, CT was not performed because the foreign body was clearly visualized on radiography, the patient was asymptomatic, and no clinical signs suggested perforation. Although spontaneous passage has been reported, prolonged retention increases the risk of mucosal injury and perforation; therefore, active removal is generally recommended when safe.

Although considered the first‐line approach, transanal manual extraction may be difficult in children because of the small anal caliber and limited cooperation. Endoscopic and surgical approaches often require general anesthesia and impose a greater physical and psychological burden on children and their families. In the present case, these considerations prompted the exploration of an alternative approach. Previous reports have emphasized a stepwise management approach for rectal foreign bodies, often progressing from manual extraction to endoscopic or surgical intervention. In contrast, the present technique offers a simple adjunct method that may obviate the need for more invasive procedures in carefully selected pediatric cases.

Button batteries are well known to cause caustic injury in the esophagus [6], but data on cylindrical batteries retained in the rectum are limited. Cylindrical batteries may theoretically cause mucosal injury through leakage or corrosion, particularly with prolonged retention. In the present case, the short rectal residence time (approximately 2 h) and absence of symptoms suggested a low risk of such complications. Magnet‐assisted extraction was considered feasible because of the metallic object. Previous studies include one adult patient in whom an electromagnet was used to remove a metallic rectal foreign body [7]. To our knowledge, this is the first pediatric report describing magnet‐assisted transanal extraction.

There are a few key points to keep in mind regarding this technique. First, it should not be attempted for sharp objects, irregularly shaped metallic items, or foreign bodies located beyond digital reach. Second, during magnet‐assisted extraction, attention should be paid to avoid trapping rectal mucosa or sphincter tissue between the magnet and object. Given that the maneuver is performed blindly, a gentle technique, an appropriately sized magnet, and readiness to abandon the attempt if resistance is encountered or if magnetic attachment cannot be achieved securely are essential. Third, postprocedural examination is recommended to assess for mucosal injury. In cases where suspicion arises that the rectal mucosa is trapped between the magnet and the foreign body during the procedure, the magnet should be left in situ without forceful manipulation, and early transfer to a pediatric referral center should be considered.

This technique may be applicable to select cases in which the foreign body is magnetic, located within digital reach, and there are no signs of perforation. This approach may reduce the need for anesthesia, endoscopy, and surgery, particularly in pediatric patients. In this case, the procedure was completed without sedation. However, intravenous sedation may be considered in patients with poor cooperation, significant anxiety, or anticipated procedural difficulty. If safe extraction cannot be achieved even with sedation, escalation to endoscopic or surgical removal under general anesthesia is recommended.

This approach should not replace established management algorithms. Rather, it should be considered an optional technique within a structured decision‐making framework. If extraction cannot be smoothly achieved, clinicians should avoid persistence and promptly proceed to the next step, such as endoscopic or surgical removal under general anesthesia. Evidence remains limited, and case series are warranted to better define the safety, efficacy, and indications of this approach.

## Author Contributions

**Daisuke Miyamoto:** conceptualization, data curation, writing – original draft. **Shunsuke Nakamura:** supervision, writing – review and editing. **Tomoki Nakamori:** supervision, writing – review and editing.

## Funding

The authors have nothing to report.

## Ethics Statement

Ethics committee approval was not required for this case report.

## Consent

Written Informed consent for publication was obtained from the patient's guardian.

## Conflicts of Interest

The authors declare no conflicts of interest.

## Data Availability

Data sharing is not applicable to this article, as no datasets were generated or analyzed during the current study.
